# From underused urban space to regenerative infrastructure: how sports parks integrate activity and sport events

**DOI:** 10.3389/fspor.2026.1979214

**Published:** 2026-10-27

**Authors:** Jin'e Yang, Baoli Zhang, Xuemo Fang

**Affiliations:** 1 School of Physical Education, South-Central Minzu University, Wuhan, China; 2 College of Physical Education and Health Sciences, Zhejiang Normal University, Jinhua, China

**Keywords:** adaptive reuse, China, community sport events, green infrastructure, physical activity, residual land, sports parks, urban regeneration

## Abstract

Sports parks offer opportunities to connect the reuse of underused urban space with everyday physical activity and sport events. This Perspective examines their regenerative potential through four illustrative cases in China: a waterfront green-space corridor in Ningbo, an industrial-heritage site in Wuhan, an expressway underspace in Ningbo, and a rooftop sports park above a rebuilt wastewater-treatment plant in Wenzhou. Drawing on site observations and publicly available project information, we interpret these cases through three connected dimensions: spatial retrofit, programmed activation, and adaptive stewardship. The cases illustrate different spatial and operating arrangements for combining land reuse, everyday recreation, and organized events. Their potential contribution to urban regeneration depends on affordable access, the compatibility of events with everyday use, and sustained maintenance and public accountability. These arrangements also raise questions about commercial pressures, uneven access, and the distribution of environmental and social benefits. We propose that sports-park policy assess land reuse alongside environmental performance, protect everyday public functions during events, and link capital investment to long-term stewardship. Further evaluation of ecological conditions and participation across social groups would clarify when these spaces contribute to inclusive and environmentally beneficial urban regeneration.

## 1 Introduction

Cities are asked to expand opportunities for physical activity while reducing land consumption and ecological fragmentation. Sports parks offer one way to address these demands by integrating opportunities for physical activity into reused waterfront spaces, former industrial sites, and infrastructure margins. In Chinese cities, the construction and retrofitting of sports facilities in these settings have become a form of urban micro-regeneration, upgrading fragmented plots through incremental interventions (1).

Facility provision, however, is only one component of urban regeneration (2). Measures such as hectares delivered, courts installed, and capital expenditure completed cannot, by themselves, establish whether a site supports sustained use or delivers social and environmental benefits. Evaluation must also consider accessibility, distributional equity, and the interests of existing communities (3). A similar distinction is needed between sport-event provision, strategic leveraging, and long-term legacy. Sustained participation depends on continued programming, institutional support, and accessible opportunities beyond individual events (4–6).

Research on recreation- and sport-led regeneration of urban water infrastructure provides a starting point for examining the relationship between spatial reuse and recreational provision (7). Smaller and non-mega events are relevant to this analysis because their outcomes depend on their integration with local organizations, facilities and communities (8). Together, these perspectives raise an integrative question: under what spatial and governance conditions can the reuse of urban space for everyday recreation and organized sport contribute to sustained public benefit? Addressing this question requires connecting the physical transformation of sites with the arrangements governing their use and maintenance.

This Perspective addresses this question through a literature-informed critical analysis of four illustrative Chinese cases. The approach brings documented spatial and operating arrangements into dialogue with research on participation, environmental justice, and stewardship, providing a basis for examining their implications where long-term outcome evidence remains limited. Using three connected analytical dimensions—spatial retrofit, programmed activation, and adaptive stewardship—we examine how spatial reuse, everyday recreation, and organized events are connected and identify the conditions that may support inclusive urban regeneration. The analysis is guided by the proposition that the regenerative contribution of sports parks depends on affordable everyday access, compatibility between events and ordinary use, and sustained stewardship.

## 2 Literature, evidence, and analytical approach

### 2.1 Literature and conceptual foundations

The analysis draws on complementary bodies of literature. A conceptual model of parks and physical activity highlights facilities, access, condition, aesthetics, safety, and policies as potentially relevant to park-based activity (9). This directs attention beyond facility provision to the conditions supporting everyday participation. Sport-event research distinguishes event delivery from sustained participation and examines how deliberate programming and organizational support can create wider community opportunities (4–6, 8). Critical urban greening research examines the distribution of environmental benefits and the relationship between public-space investment and neighborhood change (3, 10, 11). Environmental stewardship research complements these perspectives by highlighting connections among public agencies, civic organizations, and ongoing care (12).

We bring these perspectives together through three analytical dimensions. Spatial retrofit concerns the conversion of existing land and infrastructure, including its implications for access and environmental performance. Programmed activation examines the relationship between organized events, everyday recreation, and continued participation. Adaptive stewardship concerns operating responsibilities, maintenance resources, and opportunities for community influence. These dimensions guide an examination of how physical provision, patterns of use, and institutional arrangements jointly shape the regenerative potential of sports parks.

### 2.2 Case selection and evidence sources

Four cases were purposively selected to compare waterfront, former industrial, transport-infrastructure, and municipal-rooftop settings. The sites are located in Ningbo, Wuhan, and Wenzhou, with two cases sharing Ningbo's municipal context. Selection emphasizes variation in spatial and operating arrangements. Their concentration in relatively economically developed cities limits transferability to municipalities with different resources and planning conditions. Table 1 summarizes the spatial reuse, reported activation, stewardship priorities, and key management considerations across the four cases.

**Table 1:** Spatial reuse, event provision, and management considerations across four sports parks.

Case	Primary retrofit	Activation sequence & event evidence	Stewardship priority	Key management considerations
Jiangbei Riverside Sports Park, Ningbo	Continuous active-mobility and recreation network	**Event-integrated**: operator- and subdistrict-level events; 2023 marathon route node; 2025 fun run	Protect ecological continuity, daily access and temporary-event compatibility	Balance event access with everyday waterfront use
Zhangzhidong Sports Park, Wuhan	Former industrial-site conversion, heritage retention and public-space renewal	**Early programmed activation:** more than 20 mass-sport activities reported by July 2022	Sustain affordable access and accommodate heritage and informal recreation	Address affordability and barriers associated with digital booking
Panhuo Sports Park, Ningbo	Sports provision beneath an elevated expressway	**Operation-driven**: community basketball events documented in 2025	Protect free access to the public plaza and children's play areas while ensuring safety and maintenance	Balance paid sports provision with free public access and maintain service continuity
Wenzhou Sports and Leisure Park, Wenzhou	Vertically layered utility, park and sport uses	**Event-activated:** provincial U15 futsal championship; more than 20 provincial- and municipal-level football matches reported by 2023	Coordinate utility safety, public access and professional operation	Coordinate public recreation with utility operation and maintenance

The first three columns summarize case characteristics and reported activities; the final two identify management priorities informed by the analytical criteria in Section 2.

Bold text identifies the activation category assigned to each case; the text following the colon reports the available event evidence.

The evidence comprises planning and sport-administration documents, institutional project reports, media coverage, and site-based observations. Site visits to Jiangbei Riverside Sports Park, Zhangzhidong Sports Park, and Panhuo Sports Park informed the interpretation of spatial layouts and everyday uses. Descriptions of event provision draw on the cited documentary sources; the Wenzhou case relies entirely on documentary evidence. Institutional reports provide information on project delivery and activities but offer less detail on access barriers, conflict, and maintenance difficulties. Attendance and utilization figures are interpreted with attention to their reported scope and measurement detail. Figure 1 provides illustrative views of the spatial settings and recreational facilities of Jiangbei Riverside Sports Park, Zhangzhidong Sports Park, and Panhuo Sports Park.

**Figure 1:**
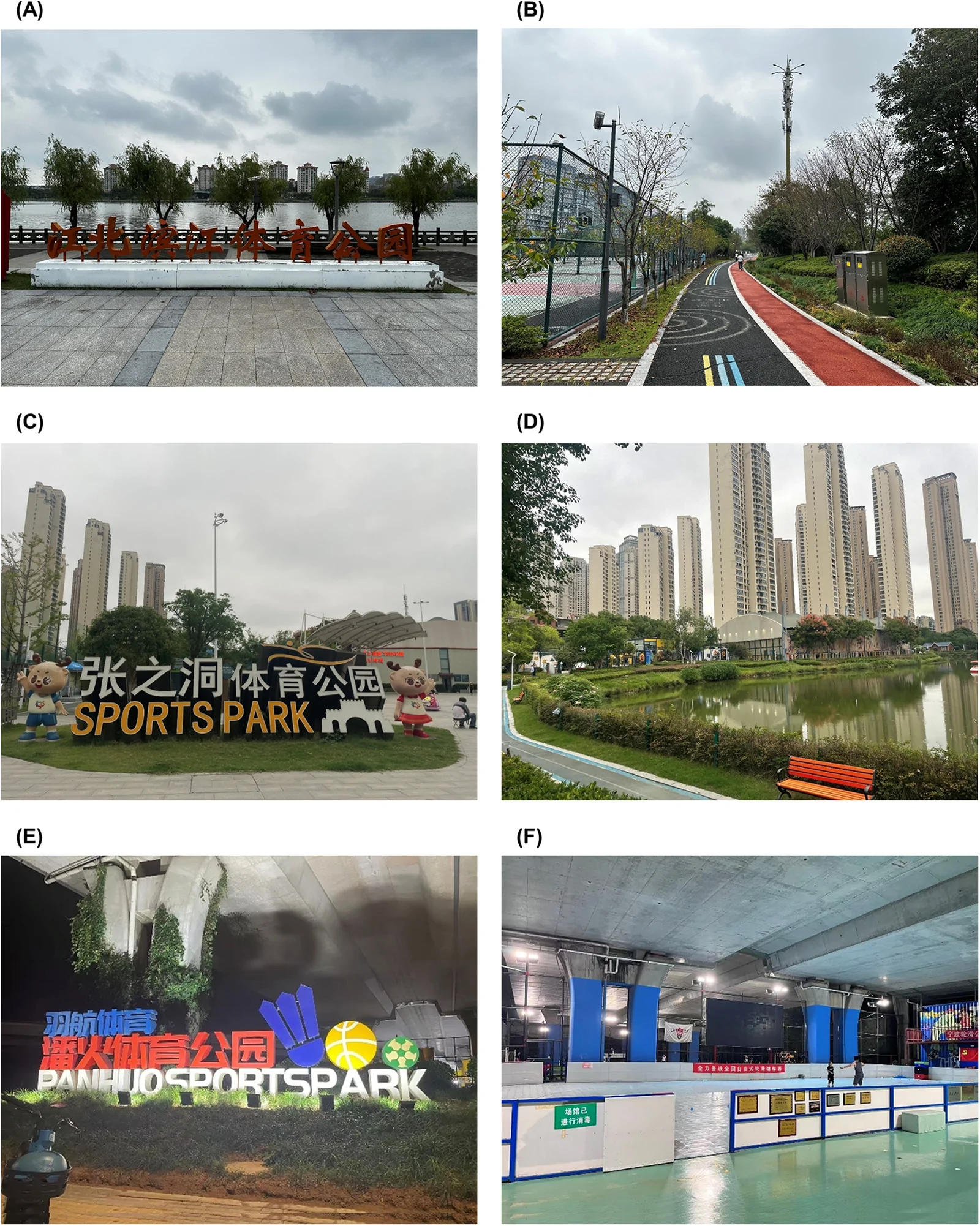
Spatial settings of three sports park cases. **(A,B)** Jiangbei Riverside Sports Park in Ningbo, Zhejiang Province: the waterfront setting and a landscaped activity path adjoining sports facilities; **(C,D)** Zhangzhidong Sports Park in Wuhan, Hubei Province: the park entrance and the integration of water, greenery, and recreational paths within the surrounding urban environment; and **(E,F)** Panhuo Sports Park in Ningbo, Zhejiang Province: the park entrance and sports facilities developed beneath elevated transport infrastructure. Photographs by Xuemo Fang and Jin'e Yang.

### 2.3 Analytical criteria and procedure

The analysis links case description, literature-informed interpretation, and cross-case comparison. Each case is examined in terms of previous land use, spatial conversion, access arrangements, reported events, and operating responsibilities. These features are considered through three questions: how spatial conversion relates to public access and environmental performance; how events share space and time with everyday recreation; and how management arrangements address affordability, maintenance, and community influence. The assessment focuses on accessible provision, compatibility between events and everyday use, and accountable arrangements for long-term care.

Critical interpretations connect specific arrangements to these questions. Paid facilities and digital booking make economic and digital accessibility relevant to the assessment of access. Assigning maintenance to an operator makes contractual responsibilities and service continuity relevant to stewardship. Cross-case comparison identifies shared concerns and conditions that depend on local institutions. Project sources provide the descriptive evidence, while the literature informs the interpretation of its implications and priorities for further evaluation.

## 3 Spatial reuse and operating arrangements in four Chinese sports parks

### 3.1 Jiangbei riverside sports park: waterfront renewal

Jiangbei Riverside Sports Park in Ningbo, Zhejiang Province, forms part of a district-wide waterfront sports-park network described in municipal sport reporting (Figures 1A,B) (13). A ‘park+sport’ co-construction policy required newly built or renovated park green spaces to allocate at least 5% of project land to public sports facilities (14). The park accommodates 16,800 m^2^ of sports facilities, 12% of its 140,000 m^2^ site, integrating running, cycling, ball games and waterfront recreation within continuous, publicly accessible green space, with differentiated everyday provision such as youth-reserved football periods (14, 15). Documented events show a multi-level continuum of integration rather than discrete modes. Reports document operator-organized activities, including a parent–child kite competition (16); sub-districts and industry organizations hold grassroots competitions and community events, including a square-dancing contest with over 200 participants (17) and a 2025 fun run for the district's construction workforce (18); and the 2023 Ningbo Marathon, with 15,000 runners, used the park as one node within a wider riverside route (19). The combination of everyday waterfront recreation and organized events makes scheduling and public access central to their compatibility. The distributional perspective introduced above also highlights the importance of examining how the benefits of waterfront investment are shared among surrounding communities.

### 3.2 Zhangzhidong sports park: industrial-brownfield renewal

Zhangzhidong Sports Park (about 52,000 m^2^) in Hanyang District, Wuhan, Hubei Province, occupies an industrial-heritage site: originally a steel-cooling pond of the Hanyang Iron Works, the land was later converted into a memorial park that fell into long-term neglect and accumulated illegal structures (Figures 1C,D) (20, 21). Its regeneration was triggered institutionally—residents' complaints during the 2018 round of central environmental-protection inspections placed the site on the priority rectification list—and the district converted it, with an investment of RMB 58 million and eight months of construction, into a smart sports park that opened in May 2021 (21). The park combines free outdoor areas with paid facilities accessed through digital booking systems (20, 21). A China News Service report republished on the municipal government website describes an estimated catchment of approximately 100,000 residents and cites the operator's figure of more than one million visits in its first year (21). By July 2022, the park was reported to have hosted more than twenty mass-sport activities, including the 9th Hanyang District National Fitness Games (21). In 2023, it hosted the two-day provincial finals of the 2nd Hubei Community Games (22). The coexistence of free outdoor areas and paid facilities accessed through digital booking makes economic and digital accessibility relevant to the assessment of public access. Long-term management also needs to accommodate informal recreation alongside organized activities and the site's historical identity.

### 3.3 Panhuo sports park: under-viaduct renewal

Panhuo Sports Park in Yinzhou District, Ningbo, Zhejiang Province, converts the underspace of an elevated expressway—a residual-land condition common in dense Chinese districts—into a multi-functional sports park stretching linearly for nearly one kilometer beneath the viaduct and covering some 9,000 m^2^, embedded in the surrounding residential fabric (Figures 1E,F). Before conversion, the plot was an overgrown, pothole-ridden leftover space: under-bridge land of this kind, with ambiguous responsibilities and high management costs, had long constituted a blind spot of urban administration. Following a systematic survey of under-bridge spaces beneath major viaducts, the district's comprehensive administrative law-enforcement bureau, noting the shortage of nearby fitness venues and after consultation with the subdistrict and communities, selected a sports-park scheme and introduced a private operator through open tendering. The operator financed the entire conversion—more than RMB 8 million over four months—and assumed responsibility for construction, operation and maintenance under an arrangement in which the right to use the site was tied to responsibility for its ongoing management (23, 24). The park combines paid sports facilities with freely accessible public areas, including a public plaza and children's play areas. Its event dimension operates at the neighborhood scale. CCTV reporting documents ‘Pan-BA’ community basketball matches at the park (25), while local reporting on the inaugural tournament in 2025 describes sixteen resident teams and collaboration among subdistrict organizations, local communities, and a corporate sponsor (26). Local reporting links the tournament to community co-governance through volunteer involvement, collaboration among communities, and community-run food stalls (26). The extent of residents' influence over programming and management would be an important focus for further assessment. Because the operator assumes construction, operation, and maintenance responsibilities, the continuity of public provision depends partly on how these obligations are specified and maintained over time. This arrangement makes affordable pricing, enforceable maintenance standards, and provision for operator withdrawal central stewardship considerations.

### 3.4 Wenzhou sports and leisure park: rooftop renewal

Wenzhou Sports and Leisure Park in Wenzhou, Zhejiang Province, incorporates sport and recreation facilities above a rebuilt semi-underground wastewater-treatment plant in the Binjiang business district (27). According to the project group's reporting, the football base had hosted more than 20 provincial- and municipal-level football matches by 2023 and had also hosted the Zhejiang Football Association youth futsal championship for boys aged under 15 (28). The project illustrates the combination of utility infrastructure and sport provision on the same site. The co-location of recreation and wastewater infrastructure makes coordination between public access and utility maintenance a central operating requirement. Evaluating its environmental contribution also requires attention to construction inputs and ongoing resource use.

## 4 Discussion

Applying the three analytical dimensions across the cases highlights how spatial provision, activity programming, and operating responsibilities interact. **Spatial retrofit** begins with what the city already has: waterfront corridors (Jiangbei), industrial structures (Zhangzhidong), bridge infrastructure (Panhuo), municipal roof decks (Wenzhou). Its first question is not “which facility is missing?” but “which urban relationship can sport help repair?” Reuse can reduce demand for additional land, although its environmental implications depend on the extent of reconstruction, material inputs, vegetation, and subsequent operation.

**Programmed activation** combines an everyday base load—accessible, low-threshold activity such as walking, informal games and social use—with periodic catalysts. Reports document organized activities soon after redevelopment at Zhangzhidong and community basketball events several years after conversion at Panhuo. In both cases, programming needs to balance event schedules with ordinary access and create opportunities for participation beyond individual events. Events add intensity, attention and organizational capacity, but these benefits do not arise automatically: they depend on deliberate leveraging strategies, responsible organizations and accessible opportunities for continued participation (5, 6). Their value should therefore be judged by what becomes recurrent or remains useful afterwards—skills, partnerships, route knowledge, equipment, public confidence. Chalip's concept of social leveraging directs attention to the organizational work needed to translate events into wider community opportunities (4).

**Adaptive stewardship** refers to the ongoing institutional capacity to adjust both spatial arrangements and programming as conditions change. It is where the cases differ most institutionally—from operating responsibilities at Panhuo to the need to coordinate utility operations and recreation at Wenzhou. Managers need a compact body of social, ecological and operating evidence. The conceptual model of parks and physical activity highlights access, condition, aesthetics, safety, and policies alongside facility provision (9). Relevant indicators include repeat use across user groups; access cost and program diversity; shade, permeability and heat exposure; maintenance response; and the reuse of temporary event assets. The purpose is not to collapse regeneration into a single score, but to reveal trade-offs and trigger adjustment. Research on urban environmental stewardship also highlights the role of civic organizations in connecting public agencies with local knowledge and ongoing care (12).

The distribution of benefits also matters. Research on green gentrification shows that new green amenities can accompany neighborhood change and unequal access to environmental improvements, with relationships varying across cities (3, 10). For sports parks, assessment should therefore consider housing affordability, participation across income groups, and the treatment of existing informal uses alongside facility provision. Paid activities and organized events may support operation while also competing with free access for space and time.

These arrangements also depend on their institutional setting. Public agencies play a central role in securing land and coordinating redevelopment in the cases examined, while commercial operators participate in service delivery. Administrative coordination can facilitate implementation, while consultation alone may give residents limited influence over final decisions on design, pricing, and programming. In settings with fragmented land ownership or greater reliance on commercial revenue, comparable projects would require negotiated access rights, enforceable service obligations, and secure maintenance arrangements. Critical research on the High Line illustrates how public-space investment can become intertwined with property development priorities (11).

Across the cases, shared-space use highlights the importance of access management, differentiated service provision raises questions of affordability, and infrastructure reuse brings long-term operating responsibilities into focus. These considerations inform four practical priorities for sports-park development and management.

First, sport-policy assessment should consider land reuse and environmental performance alongside facility provision, with claims of ecological repair supported by measured environmental indicators. Relevant indicators include retained structures, surface permeability, and measured changes in environmental conditions.

Second, event-specific facilities should not permanently displace the everyday public functions of sports parks. Routes, barriers, signage and spectator facilities should be temporary, removable or adaptable, and event plans should explain how ordinary access will be protected before, during and after each event.

Third, assessment frameworks for sports parks should distinguish between built capacity, activation and legacy. Provision alone does not guarantee intended patterns of use: observational research has shown that some park facilities attract users who differ from their intended target groups (29). Assessment should combine patterns of use with affordability, user diversity, and environmental conditions.

Fourth, capital funding must be paired with stewardship capacity. Residual spaces commonly cross departmental boundaries, and green and sport assets have different maintenance cycles. A named coordinating body, multi-year operating budget and community feedback mechanism are as important as design quality. Public–private or event revenue can help, but free access should not become contingent on commercial programming. Operating agreements should specify maintenance standards, affordable access, and responsibilities if revenue declines or an operator withdraws.

The analysis is limited by its concentration in three relatively economically developed cities and by the reliance on institutional reporting. The available materials contain no direct measurements of ecological change, and provide limited information on participation across social groups, affordability, and maintenance over time. Future research could combine repeated observations, resident perspectives, operating records, and environmental measurements across municipalities with different resources and planning arrangements.

## 5 Conclusion

Sports parks offer a means of bringing everyday recreation and organized sport into the reuse of urban space. The four cases illustrate how waterfront, former industrial, transport, and utility settings accommodate different spatial and operating arrangements. Their contribution to urban regeneration depends on more than facility provision: affordable access, compatibility between events and ordinary use, and sustained maintenance shape who benefits and how these spaces function over time. The three dimensions of spatial retrofit, programmed activation, and adaptive stewardship provide a basis for examining these relationships across planning contexts. Connecting project delivery with evaluation of social and environmental conditions would strengthen sports parks' role in inclusive urban regeneration.

## Data Availability

The evidence discussed in this Perspective consists of publicly available documentary sources cited in the reference list and site-based observations by the authors.
